# Self-Regulation of Brain Activity in Patients with Postherpetic Neuralgia: A Double-Blind Randomized Study Using Real-Time fMRI Neurofeedback

**DOI:** 10.1371/journal.pone.0123675

**Published:** 2015-04-07

**Authors:** Min Guan, Lijia Ma, Li Li, Bin Yan, Lu Zhao, Li Tong, Shewei Dou, Linjie Xia, Meiyun Wang, Dapeng Shi

**Affiliations:** 1 Department of Radiology, People’s Hospital of Zhengzhou University, Zhengzhou, Henan, China; 2 Department of Dermatology, Second People’s Hospital of Zhengzhou, Zhengzhou, Henan, China; 3 China National Digital Switching System Engineering and Technological Research Center, Zhengzhou, Henan, China; 4 McConnell Brain Imaging Center, Montreal Neurological Institute, McGill University, Québec, Canada; 5 Department of Pain Management, People’s Hospital of Zhengzhou University, Zhengzhou, Henan, China; Yale University, UNITED STATES

## Abstract

**Background:**

A pilot study has shown that real-time fMRI (rtfMRI) neurofeedback could be an alternative approach for chronic pain treatment. Considering the relative small sample of patients recruited and not strictly controlled condition, it is desirable to perform a replication as well as a double-blinded randomized study with a different control condition in chronic pain patients. Here we conducted a rtfMRI neurofeedback study in a subgroup of pain patients – patients with postherpetic neuralgia (PHN) and used a different sham neurofeedback control. We explored the feasibility of self-regulation of the rostral anterior cingulate cortex (rACC) activation in patients with PHN through rtfMRI neurofeedback and regulation of pain perception.

**Methods:**

Sixteen patients (46–71 years) with PHN were randomly allocated to a experimental group (n = 8) or a control group (n = 8). 2 patients in the control group were excluded for large head motion. The experimental group was given true feedback information from their rACC whereas the control group was given sham feedback information from their posterior cingulate cortex (PCC). All subjects were instructed to perform an imagery task to increase and decrease activation within the target region using rtfMRI neurofeedback.

**Results:**

Online analysis showed 6/8 patients in the experimental group were able to increase and decrease the blood oxygen level dependent (BOLD) fMRI signal magnitude during intermittent feedback training. However, this modulation effect was not observed in the control group. Offline analysis showed that the percentage of BOLD signal change of the target region between the last and first training in the experimental group was significantly different from the control group’s and was also significantly different than 0. The changes of pain perception reflected by numerical rating scale (NRS) in the experimental group were significantly different from the control group. However, there existed no significant correlations between BOLD signal change and NRS change.

**Conclusion:**

Patients with PHN could learn to voluntarily control over activation in rACC through rtfMRI neurofeedback and alter their pain perception level. The present study may provide new evidence that rtfMRI neurofeedback training may be a supplemental approach for chronic clinical pain management.

## Introduction

Postherpetic neuralgia (PHN) is a common type of neuropathic pain characterized as a sharp, burning or stabbing pain following herpes zoster infection [1]. It can profoundly affect the quality of life of suffers in terms of loss of productivity and associated cost implications [2]. Moreover, such a lasting chronic pain may increase the risk of depression and suicide [3]. Patients with PHN are always treated with pharmacological interventions over a long time and the cumulative drug-related side effects may pose a considerable risk. Thus, new supplementary therapies that could generate improved pain relief are expected.

Biofeedback is one of the most prominent behavioral approaches in which patients learn to voluntarily control over their bodily reactions through the feedback of physiological processes, such as brainwaves, muscle tone, skin conductance, heart rate and pain perception [4]. Neurofeedback (mainly based on Electroencephalography (EEG)) is a type of biofeedback that provides individuals with real-time feedback of different physiological parameters to develop awareness of and to control over specific behavior [5]. It has been applied to manage diverse pain syndromes, e.g. phantom limb pain [6], complex regional pain syndrome (CRPS) [7], fibromyalgia syndrome [8]. However, the lack of precise localization and limited coverage of EEG pose a challenge on providing accurate feedback of the activity of localized brain areas [9]. Real-time functional magnetic resonance imaging (rtfMRI) neurofeedback is a new emerging technology that combines rtfMRI and neurofeeback. RtfMRI enables blood oxygen-level-dependent (BOLD) fMRI data processing and displaying concomitantly with image acquisition, and neurofeedback allows a subject to watch and regulate the fMRI signal from his/her brain [10]. Compared with EEG-neurofeedback, rtfMRI neurofeedback can precisely locate brain regions for a specific regulation. It has been applied to various studies on mood regulation [11], language processing [12], rehabilitation after stroke [13], chronic tinnitus [14] as well as pain management [15].

The first rtfMRI neurofeedback study concerning pain modulation demonstrated that individuals could gain voluntary control over activation in the rostral anterior cingulate cortex (rACC), which was putatively involved in pain processing, leading to control over pain perception [15]. However, regarding the clinical utility of rtfMRI neurofeedback procedures, two limitations of this study needed to be taken into account: First, the sample size of patients included in the experimental group was relatively small (only 8 patients with chronic pain, such as complex regional pain syndrome and fibromyalgia). Thus, replication would be of great value for verifying the effect of rtfMRI neurofeedback [16]. Second, this study was not strictly controlled for pain condition. The patients in the control group were given autonomic neurofeedback (e.g. skin conductance, heart rate or respiration rate) other than feedback information from their brains. In addition, the finding of this study could not be replicated by deCharms et al. in a later follow-up study; as publicly stated at the rtfMRI conference in Zurich, 2012 [17]. Thus, a randomized controlled trial may be needed for exploring the definite therapeutic effect of rtfMRI neurofeedback on pain management.

In this work, we aimed to explore whether patients with PHN were able to up and down regulate their rACC BOLD signal through rtfMRI neurofeedack, and whether the regulation would alter pain perception level. We predicted that our results would be consistent with previous rtfMRI studies concerning pain management [15, 18–19]. Especially, we used a different patient control condition, in which patients were given neurofeedback information from their own brain regions. This kind of control condition has been shown to be helpful for identifying the specific modulation effect of rtfMRI neurofeedback [20]. We investigated whether the neurofeedback from rACC is related to the pain control enhancement within this region; whether changes in perception were caused by the feedback from rACC or by other placebo effects.

## Materials and Methods

### Subjects

The study was conducted in accordance with the principles of Declaration of Helsinki [21] and approved by the ethics committee of People’s Hospital of Zhengzhou University. All subjects gave written informed consent to participate in the study.

16 right-handed patients with PHN were enrolled from the Pain Management Department of People’s Hospital of Zhengzhou University, China. The diagnosis was made by a senior doctor in pain management (LJ.X) according to the International Association for the Study of Pain (IASP) criteria [22]. Exclusion criteria included: (1) any major neurological or psychiatric disorders; (2) exposure to any medication likely to influence cerebral function or blood flow within 1 month; (3) history of head trauma; (4) drug or alcohol abuse; (5) any MRI contraindication; (6) duration of self-reported chronic spontaneous PHN pain was less than 1 month.

A randomized, double-blind, sham-controlled between-subject design was utilized. (1) Experimental group: 8 patients (5 males, 3 females) were given true feedback information from their rACC BOLD signal. (2) Control group: 8 patients (4 males, 4 females) received identical training to the experimental group, but using rtfMRI information derived from the posterior cingulate cortex (PCC). PCC has been implicated as an area not involving in pain processing [15]. All patients, and all staff involved were blind to group assignment, except the principal investigator who was not involved in data-collection, data-entry and data-analysis (DP. S).

### Experimental paradigm

**Fig 1:**
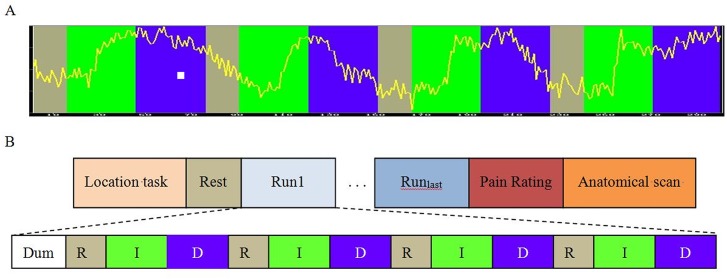
Design of the rtfMRI neurofeedback experiment. A) a scrolling line graph representing BOLD activation shown to patients during scanning. They were instructed to increase the line within the green stage, decrease the line within the blue stage, and keep quiet during gray stage. B) Paradigm of the rtfMRI nurofeedback study. The experimental protocol was consisted of a localizer task, rest, rtfMRI neurofeedback training, pain rating and anatomical scan. During a training run, patients underwent rtfMRI neurofeedback training consisted of alternating blocks of Rest (R, gray block), Increase (I, green block), and Decrease (D, blue block), lasting 30, 60, 60 seconds respectively.

Before MRI scanning, all patients were given a detailed explanation about the purpose of the study. Then a sample scrolling line graph representing BOLD activation (the same as real neurofeedback) was shown and explained to them clearly (Fig 1A). The patients were instructed to up and down regulate activation of their brain regions using certain cognitive strategy during MRI scanning. According to the first international rtfMRI neurofeedback conference in Zurich, 2012, a well-known explicit strategy would be efficient for self-regulation [16]. Here, we gave certain instructions including changing attention or stimulus severity. Explicit examples were also given, which included imagining happy moments such as gaining money or family time (changing attention) or some pain condition such as stabbing, burning or electric shock (stimulus severity). In case of a successful regulation, the patient would see the scrolling line up or down according to the corresponding colour.

The paradigm was consisted of two fMRI tasks (Fig 1B). First, a typical block-designed localizer task was conducted for drawing rACC manually as precisely as possible. It was composed of five 30 seconds task block interspersed with five 30 seconds rest blocks. For ethical issues, during the task blocks a handmade painful stimulus was given on the skin of the right wrist of each patient by a medical bachelor student (XN. Z) from Zhengzhou University. Before the stimuli, she had a conversation with each patient and practiced to elicit a constant painful sensation using an intensity that was manageable for the subject. This would make sure that a proper stimulus was given without any side effect. Before the first block, a dummy scan lasting 6 seconds was conducted to make sure the MRI scanner and the participant reach a stable state. Second, rtfMRI neurofeedback training task was conducted, during which the patients were instructed to regulate BOLD activation of target brain regions. An uprated scrolling line graph of BOLD signal from entire target regions of interest (ROIs) (rACC in the experimental group and PCC in the control group) was projected to the subjects in real-time. Each training run consisted of a 30 seconds rest block, followed by a 60 seconds increase block during which the subjects were trained to increase ROI activation, and a 60 seconds decrease block during which subjects were trained to decrease ROI activation. Each patient underwent a series of training runs. For ethical considerations, the scanning termination was determined by the patients themselves.

### ROI localization

**Fig 2:**
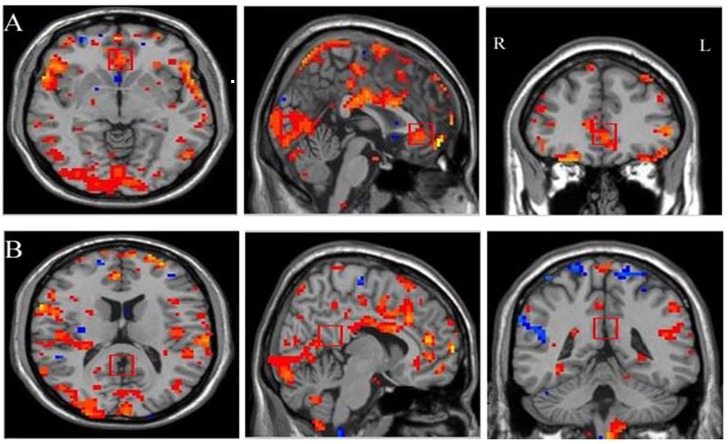
Example of Regions of Interest (ROI) selection for rtfMRI neurofeedback training. Two squared regions of interest (1 cm per side) were placed for acquiring scrolling line graph. A) rACC was selected based on the activation observed in the localization task. B) PCC was selected according to the anatomic expertise of a senior radiologist. Following radiological notation, the left side (L) of the brain is shown on the right, and the right side (R) of the brain is on the left.

ROIs for the neurofeedback were individually selected during the localizer task based on the activation observed within rACC using the pain—rest contrast. Then, a squared ROI with the side length of 1 cm in a single slice was positioned manually (Fig 2A). Such ROI selection may be more robust to small subject movement or statistical fluctuations in activation patterns than a smaller or thresholded cluster defined by statistical criteria [15, 19]. PCC was selected on T1 images based on the anatomic expertise of a senior radiologist (M. G) (Fig 2B).

### Pain rating

To quantify the pain perception level of each patient and to assess the therapeutic effect of the rtfMRI neurofeedback, a 11—point numerical rating scale (NRS) ranging from 0 (not painful) to 10 (the most painful) was adopted [23]. The patients were asked to report their NRS before MRI scanning and after the last training run (for up and down regulation separately).

### Data acquisition

MR imaging was conducted at the Medical Imaging Center of People’s Hospital of Zhengzhou University. The rtfMRI neurofeedback system was composed of a 3.0T MRI scanner (Discovery 750, GE Healthcare, Milwaukee, USA), Turbo-Brain Voyager (TBV) Version 1.1 (Brain Innovation, Maastricht, The Netherlands) and a Visual Stimulation System for fMRI (Sinorad, ShenZhen, China) (S1 Fig). An in-house program written in C and Python was used for fMRI data transferring in real-time.

A standard 8-channel birdcage head coil was used, along with restraining foam pads to minimize head motion and to diminish scanner noise. MR scanning was performed in the following order: (1) A localizer task-fMRI scanning using GRE-T2* EPI sequence. The following EPI imaging parameters were: TR (repetition time) = 2000 ms, TE (echo time) = 30 ms, slice thickness = 4.0 mm with the interslice gap of 0 mm, slices = 33, field of view = 220 × 220 mm^2^, matrix size = 64 × 64, flip angle = 90°, number of volumes = 153. (2) the rtfMRI neurofeedback training run using the same sequence and parameters as the localizer task, except the number of volumes was increased to 303. (3) A high-resolution anatomical scan was acquired for image normalization using a three dimensional fast spoiled gradient (3D FSPGR) sequence. The parameters were: TR = 8.2 ms, TE = 2.26 ms, in-plane matrix resolution = 256 × 256, slices = 200, field of view = 256 × 256 mm^2^, flip angle = 12°.

### Data processing and statistical analysis

#### (1) Online analysis

Functional imaging data was preprocessed and statistically analyzed on-line using TBV within one TR [24]. Preprocessing of the data included incremental linear detrending, 3D motion correction and spatial smoothing using a Gaussian kernel with the full width half maximum (FWHM) of 8 mm. A recursive least squares regression algorithm was used for correlation analyses. For the linear detrending, a linear predictor was added as a confounding to the Generalized Linear Model (GLM). Statistical analysis was implemented based on the GLM with the software.

#### (2) Offline analysis of the functional localizer

Off-line data analysis was performed with Statistical Parametric Mapping (SPM 8) (http://www.fil.ion.ucl.ac.uk/spm). Functional data was spatially realigned, co-registered to the anatomical data, normalized and smoothed (8 mm kernel). Then group analysis was performed based on GLM using the block design. Family-wise error (FWE) corrected values of p<0.05 were considered as significant.

#### (3) Offline analysis of the ROI activation of the neurofeedback training runs

ROI analysis was performed with the Analysis of Functional NeuroImages (AFNI) analysis package (http://afni.nimh.nih.gov/). As we were only concerned about the final modulation effect of rtfMRI neurofeedback, only the first and last training run of each patient was selected for the signal change analysis. The rate of BOLD signal change *p* was computed as:

$$p=\frac{\left(block-mean\right)}{mean}\times 100\%$$ (1)

where *block* is the average signal intensity of the task block which included all up-regulation or down-regulation blocks, *mean* is the average signal intensity of the base contrast [15]. The signal change between the last and first training run Δ*p* was calculated as Δ*p* = *P*_last_-*P*_first._ Two–tailed one-sample t-tests were conducted to assess the difference of Δ*p* comparing with 0 during up and down regulation separately. One-tailed two-sample t-tests were conducted to assess the difference of Δ*p* between the experimental and control groups. All tests were conducted using SPSS 17.0 (SPSS Inc, Chicago, IL, USA). The critical threshold for significance was set at p<0.05.

#### (4) Correlation Analysis

To explore whether BOLD signal change of rACC was correlated with patients’ pain perception levels, the association between Δp and NRS changes during up or down regulation was determined using Spearman’s Rho. The threshold for significance was set at p < 0.05.

## Results

Two patients (1 male and 1 female) in the control group were eliminated due to the large head motion. Finally, data from 14 patients (8 in the experimental group, 6 in the control group) were analyzed. 10 patients completed two training runs, 2 patients completed three training runs, and 2 patients completed four training runs. Both groups did not differ in gender proportion, mean age, duration of PHN, NRS baseline score and training runs (Table 1).

**Table 1:** Demographic characteristics of Experimental and Control group.

	**Experimental group (n = 8)**	**Control group (n = 6)**	***P* value**
Gender(male/female)	5/3	3/3	>0.99
Mean age(years)	58.50 (2.24)	61.33 (3.43)	0.49
Duration(months)	4 (0.73)	5 (1.16)	0.6
NRS baseline	4.13 (0.55)	5.00 (0.52)	0.28
Training runs	2.38 (0.26)	2.50 (0.34)	0.77

Numbers in parentheses indicate standard error of the mean (SEM). P values refer to Fisher's exact test for proportion of females and two-sample t tests for other characteristics.

Abbreviations: NRS, numerical rating scale.

In addition, to assess the difference of pain localization in both groups, the pain regions complained by each patient was classified into 4 main anatomical regions: that craniofacial region, cervix and upper limb, thorax, abdomen. Fisher's exact test was performed, and the result showed that both groups did not differ in the distribution of pain location (P > 0.05).

### Online analysis

**Fig 3:**
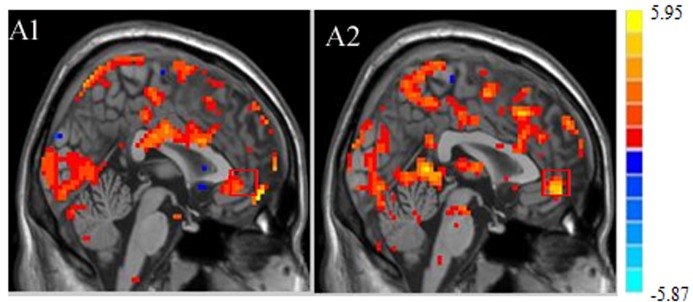
The brain activation maps of rACC by on-line analysis in one patient. Brain activation of rACC during up regulation in the first training run (A1) and last training run (A2). A stronger activation can be seen in last training run, indicating a successful training.

Through the course of training, 6/8 patients in the experimental group were able to learn to control the fMRI activation in their own rACC. An uprated line of BOLD activation went regularly up and down as the study design (Fig 1A). Accordingly, the activation of rACC for the last training was stronger than the first one (Fig 3).

### Offline analysis of the functional localizer

The functional localizer revealed significant activation within the rACC, the Basal Ganglia (BG), the Inferior Frontal Gyrus (IFG), the Premotor Cortex (PMC), the Inferior Temporal Gyrus (ITG), the Superior Temporal Gyrus (STG), and the precuneus. All these regions were involved in pain processing (S1 Table).

### Offline analysis of the ROI activation

**Fig 4:**
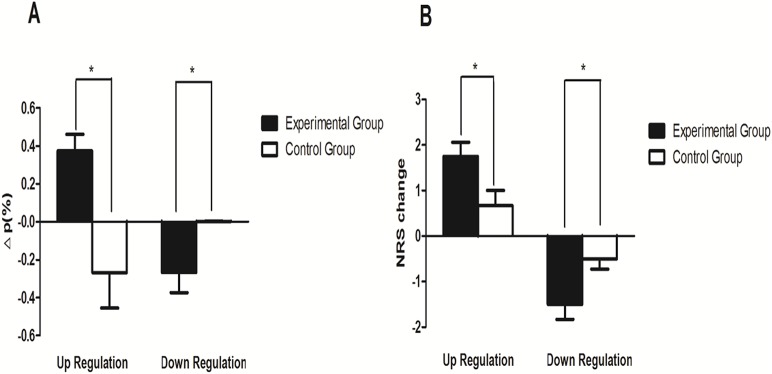
Results of off-line ROI analysis and NRS change. Mean BOLD signal change (Δp) and NRS change during up and down regulation between experimental and control group were compared. The symbol * indicates the difference between the two groups is significant at the 0.05 level. Values are mean ± standard error of the mean (SEM).

The difference of the percent signal change (Δ*p*) between the last and first training runs was assessed using two tailed one-sample t-tests (comparing activation to 0). The values of Δ*p* during up and down regulation were both significantly different than 0 (t(7) = 4.259; p < 0.01 for up regulation; t(7) = -2.51; p < 0.05 for down regulation). Also there was a significant Δ*p* between experiment group and control group (t(12) = 3.39; p < 0.01 for up regulation; t(12) = 7.01; p < 0.05 for down regulation). In control group, no significant difference of Δ*p* between the last and first training runs was observed during up regulation (t(5) = -1.44; p > 0.05) and down regulation (t(5) = 0.51; p > 0.05) (Fig 4A). These results suggested that only patients in the experimental group were able to significantly regulate BOLD activity, and this effect was mainly limited to rACC.

### Correlation analysis

The mean change of NRS for both groups was listed in Table 2. For the experimental group, significant change of NRS was both seen in up regulation (t(12) = -2.34; p < 0.05) and down regulation (t(12) = 2.34; p < 0.05) (Fig 4B). Finally, we tested whether there was an association between Δp and NRS changes. However, there existed no significant correlations (r = 0.16; p = 0.26 for up regulation; r = 0.27; p = 035 for down regulation).

**Table 2:** Change in BOLD signal of rACC and NRS following neurofeedback training for both group.

	**Experimental group** (n = 8)	**Control group** (n = 6)	***P* value**
Δ**p up-regulation**	0.37(0.09)	-0.27(0.19)	0.005
Δ**p down-regulation**	-0.27(0.11)	0.002(0.003)	0.039
**NRS change for up-regulation**	1.75(0.31)	0.67(0.33)	0.038
**NRS change for down-regulation**	-1.50(0.33)	-0.50(0.22)	0.037
	**Experimental group (n = 8)**	**Control group (n = 6)**	***P* value**
Δp up-regulation	0.37 (0.09)	-0.27 (0.19)	0.005
Δp down-regulation	-0.27 (0.11)	0.002 (0.003)	0.039
NRS change for up-regulation	1.75 (0.31)	0.67 (0.33)	0.038
NRS change for down-regulation	-1.50 (0.33)	-0.50 (0.22)	0.037

Numbers in parentheses indicate standard error of the mean (SEM). P values refer to the two-sample t tests.

Δp refers to the signal change between the last and first training run of rACC for the experimental group and PCC for the control group.

Abbreviations: NRS, numerical rating scale.

## Discussion

In this study, we investigated the feasibility of training patients with PHN to learn to increase and decrease the level of activation of rACC using rtfMRI neurofeedback. The results demonstrated that given appropriate directed training based on rtfMRI neurofeedback information, patients with PHN could learn to up and down regulate the BOLD activity of rACC, leading to alter their pain perception within a short time of training. Moreover, using a strict control condition, our results demonstrated that this training effect of rtfMRI neurofeedback was due to the target rACC region other than a placebo effect. Our study may provides new evidence that rtfMRI neurofeedback training may be a possible supplemental approach for pain management.

### Control over rACC activation

It has been widely accepted that pain was a brain-based phenomenon [25]. Neuroimaging studies have identified a pain modulatory system, which is consisted of ACC, the primary and secondary somatosensory areas (SI, SII), the primary motor (MI) and the PMC, the supplementary motor area (SMA), the BG, the parietal and insular cortices, the periaqueductal gray (PAG), the rostral ventromedial medulla, the hippocampus, the amygdala, the parahippocampus, and the prefrontal cortex (PFC) [26]. Dysregulation of this modulatory system may underlie chronic pain condition [27]. Thus, it is a reasonable hypothesis that directly manipulating brain regions could enhance pain modulatory systems and thereby could ultimately reverse the abnormalities underlying chronic pain [28]. In this study, we found that given appropriate explicit strategies and rtfMRI neurofeedback training, the BOLD signal change of rACC in the experimental group was significantly different than 0 and was significantly different from the control group during up and down regulation respectively. This indicates that patients with PHN in the experimental group could learn to regulate their rACC activations through rtfMRI neurofeedback training. This is consistent with the first rtfMRI study reported [15, 29]. In a recent study, Rance et al. [18] found that healthy adults could successfully learn to decrease rACC activation in response to the painful stimulus. However, in that study, the participants failed to up regulate rACC activation. This may be due to the different subjects recruited and experimental paradigms. In addition, the rACC activation has been found increased in the generation of negative emotion [30], thus it may be more difficult for healthy subjects to up regulate rACC than the PHN patients.

The online analysis on TBV showed that 2 out of 8 patients in the experimental group did not succeed in regulation. This was consistent with one study exploring the effect of training in chronic tinnitus, in which 1 of 6 patients could not succeed [14]. In a similar pain study, Emmert et al. found 8/14 subjects could down regulate the rACC activation [19]. This may be due to the unconcentration state of the patient inside a MRI scanning environment or an ineffective cognitive strategy used.

### Mental strategies for regulation

Choosing an efficient mental imagery strategy for self-regulation is challenging for rtfMRI neurofeedback training. Earlier conventional wisdom suggests that providing a well-known explicit strategy to the subject would enable more efficient self-regulation, and that implicit mental imagery strategies would be time-consuming in the harsh and expensive MR environment [16]. Hence we used an explicit strategy including changing attention or controlling stimulus severity. We found 6/8 patients in the experimental group were able to learn to control the fMRI activation of their rACC. This result illustrated that these strategies were useful for self-regulation. This may be due to the fact that the patients with PHN in our study have endured chronic pain for such a long time and are experienced at using mental strategies to reduce pain perception in their daily lives.

### rACC signal change and pain perception

Several previous studies have shown that learned control of brain activity would lead to behavioral effects that are specific to the functional role of the target brain area [31, 32]. deCharms and Colleagues found that after rtfMRI nerurofeedback training, all chronic pain patients reported a decrease in their pain, and 5 of 8 reported reductions in pain of 50% or more using the McGill pain questionnaire [15]. Our study found that after the rtfMRI neurofeedback training, the pain perception level for down regulation measured as NRS in the experiment group reduced 1.5, which was significantly greater than the one in the control group. The different level of pain perception reduction of the former study and ours may be due to the different severities of the patient recruited and the different scales used. In addition, we did not find a significant correlation between the percent BOLD signal change rate and the resultant NRS changes. This may be due to the fact that simple regulation of one single multifunctional node within a wider pain network does not lead to a significant change in the perception of pain intensity [33]. In addition, our relatively small sampl size might not have enough statistical power to detect the significant correlations.

### Possible mechanisms underlying rACC regulation and resultant NRS changes

The mechanism underlying self-regulation and corresponding behavior change has been unclear. Until now, few studies have tested specific theoretical hypotheses about the mechanism of operant and cognitive control of neural activity. At the brain level, rACC is a specific area linked to attention [34], emotion [35], saliency [36] and self-regulation [37], all of which are relevant to pain. ACC is often suspected to be of paramount importance for the development of chronic pain due to its integrative function [38]. Functional neuroimaging studies have shown that ACC could be activated by focused attention to noxious stimuli, whereas the effect was opposite while distraction from the noxious stimuli [39]. Thus, it is rational that regulation of activity of rACC may play a role in stimulation-induced pain relief. At the neuron level, increased synaptic activity in the rACC/orbitofrontal cortex may be an important common effect of both drug and neuro-stimulation analgesia, reflecting presumably increased activity in pain control areas [40]. In addition, induced neuronal activation would lead to consolidation or strengthening of the used connections and networks, perhaps through Hebbian mechanisms of learning [41]. Future studies of rtfMRI neurofeedback combining both BOLD and electrophysiological measurements might help explore the mechanisms at both levels.

### Real neurofeedback OR sham neurofeedback

As many confounding factors may affect the effect of rtfMRI neurofeedback training, selecting an optimal control task is essential for determining whether the feedback signal is necessary for learning to regulate brain activation, compared to the effects on brain activation produced through repetitive training using simple instructions alone [15]. However, there exists no common criterions of an ideal control condition for neurofeedback study. Four kinds of control conditions were usually adopted by existing studies: (1) control groups received sham feedback derived from other participants' data or artificially created [12, 42, 43]; (2) regulation without the feedback [15]; (3) neurofeedback received autonomic biofeedback information [15]; (4) neurofeedback received contingent feedback (i.e. directly related to the feedback signal) from areas other than the experimental target region [13,15, 20, 44]. The last choice was believed to be the best suited for determining the specificity of neurofeedback training, such as, whether feedback from the target region is necessary for enhanced control of that region; and whether changes in behavior performance are due to feedback from the target region or due to a placebo effect [16]. Therefore, our study adopted a sham neurofeedback from PCC as patient control condition. The experimental and control group were the same in clinical severity, demographic data and MRI procedure, except for that the experimental group received information from rACC while the control group received information from PCC. We found that only in the experimental group, the patients could learn to regulate rACC successfully and their pain perception changed significantly, suggesting that the feedback information from a specific brain region is necessary for rtfMRI neurofeedback regulation and the behavior changes [18–19].

### Limitations and future direction

The following limitations should be taken into account when interpreting the current results. First, due to the small sample size in this work, the present results should be interpreted with caution. It is desirable to replicate the study with larger patient samples and other pain conditions, although the requirement of a good and voluntary corporation for rtfMRI neurofeedback is a challenge for recruiting more patients for the study. Second, a transfer run was not included for the ethical consideration and to focus on regulation training. Next we will conduct a study exploring the lasting effect of rtfMRI neurofeedback with such a design. Third, the complex confounding effects of PCC as the control region should be reconsidered in our study. On the one hand, unsuccessful sham control may induce frustration or other negative emotion. Thus more cognitive or emotional processes may be involved and interfere with the regulation. On the other hand, PCC is one of the key nodes in the default mode network (DMN) and usually deactivated during cognitively tasks. It may prevent the PHN patients from successfully up-regulating brain activation. Thus it is meaningful to conduct a study selecting other region like the centrum ovale. Fourth, a manual stimuli delivery was adopted for functional localizer task, there might be a certain amount of variability between stimulation blocks. Last, the individual training runs in our study were not identical, and only the first and the last runs were analyzed. The difference in training may confound the true effect of neurofeedback. As a matter of fact, we are looking for a chance to conduct a more rigorous study investigating the correlations between the regulation effects and training runs.

## Conclusions

In conclusion, our study demonstrates that patients with PHN could voluntarily regulate activity of their rACC through rtfMRI neurofeedback training. As a result, their pain perception changed significantly. In contrast, patients with PHN in control group receiving neurofeedback information from PCC could not control over rACC and pain perception level. Our study suggests that rtMRI neurofeedback training may be a supplemental approach for pain relief of chronic clinical pain in short time.

## Supporting Information

S1 Fig Display of rtfMRI neurofeedback system and TBV interface. A) The framework of rtfMRI neurofeedback system. The system was composed of a 3.0 T MRI scanner, data processing workstation and a displaying device. B) The interface of Turbo-Brain Voyager (TBV). The scrolling line of ROI in the top right was displayed to participants in scanner. The head motion parameters in the down right may help control the quality of fMRI data.
(TIF)

S1 Table Activation during painful stimulation in the functional localizer task. H = hemisphere, R = right hemisphere, L = left hemisphere, rACC = rostral anterior cingulate cortex, BG = Basal Ganglia, IFG = Inferior Frontal Gyrus, PMC = Premotor Cortex, ITG = Inferior Temporal Gyrus, STG = Superior Temporal Gyrus, TAL = Talairach coordinates, all values were family-wise error (FWE) corrected (P < 0.05).
(DOCX)
