# Supplementary figures and images for: Self-Regulation of Brain Activity in Patients with Postherpetic Neuralgia: A Double-Blind Randomized Study Using Real-Time fMRI Neurofeedback
Source: PLoS One. 2015 Apr 7;10(4):e0123675. doi: 10.1371/journal.pone.0123675 (PMC4388697; doi:10.1371/journal.pone.0123675)

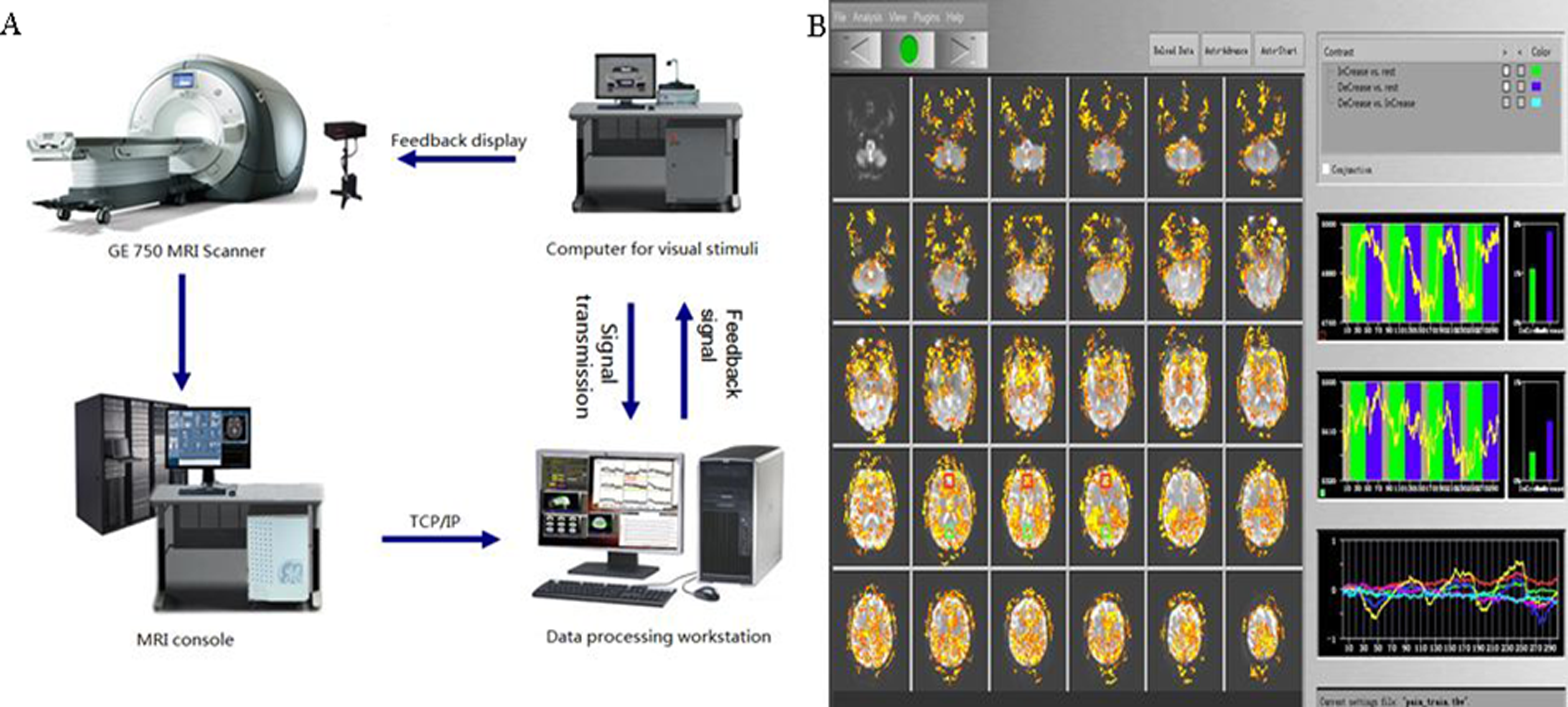

Supplement: S1 Fig — A) The framework of rtfMRI neurofeedback system. The system was composed of a 3.0 T MRI scanner, data processing workstation and a displaying device. B) The interface of Turbo-Brain Voyager (TBV). The scrolling line of ROI in the top right was displayed to participants in scanner. The head motion parameters in the down right may help control the quality of fMRI data. (TIF) [file pone.0123675.s001.tif]
