# Supplementary material for: Self-Regulation of Brain Activity in Patients with Postherpetic Neuralgia: A Double-Blind Randomized Study Using Real-Time fMRI Neurofeedback
Source: PLoS One. 2015 Apr 7;10(4):e0123675. doi: 10.1371/journal.pone.0123675 (PMC4388697; doi:10.1371/journal.pone.0123675)
Supplement: S1 Table — H = hemisphere, R = right hemisphere, L = left hemisphere, rACC = rostral anterior cingulate cortex, BG = Basal Ganglia, IFG = Inferior Frontal Gyrus, PMC = Premotor Cortex, ITG = Inferior Temporal Gyrus, STG = Superior Temporal Gyrus, TAL = Talairach coordinates, all values were family-wise error (FWE) corrected (P < 0.05). (DOCX) [file pone.0123675.s002.docx]

**Table SI: Activation during painful stimulation in the functional localizer task.**

| Function Localizer task | |  |  |  | TAL | | |
| --- | --- | --- | --- | --- | --- | --- | --- |
| lobe | H | structure | *t-*value | *p-*value | x | y | z |
|  |  |  |  |  |  |  |  |
| frontal | L | rACC | 5.50 | < 0.05 | 0 | 36 | 9 |
|  | L | BG | 6.80 | < 0.05 | 24 | -48 | 21 |
| frontal | R | IFG | 6.50 | < 0.05 | 43 | 33 | -6 |
| frontal | R | PMC | 6.38 | < 0.05 | 63 | 0 | 27 |
| temporal | R | ITG | 4.37 | < 0.05 | 42 | -6 | 39 |
| temporal | R | fusiform gyrus | 3.49 | < 0.05 | 54 | -39 | -21 |
| temporal | R | STG | 3.1 | < 0.05 | 57 | -39 | 12 |
| parietal | L | Precuneus | 3.01 | < 0.05 | -18 | -60 | 48 |

H = hemisphere, R = right hemisphere, L = left hemisphere, rACC = rostral anterior cingulate cortex, BG = Basal Ganglia, IFG = Inferior Frontal Gyrus, PMC = Premotor Cortex, ITG = Inferior Temporal Gyrus, STG = Superior Temporal Gyrus, TAL = Talairach coordinates, all values were family-wise error (FWE) corrected (P < 0.05).
